# Analyses of the effectiveness of a Brazilian pediatric home care service: a preliminary study

**DOI:** 10.1186/s12913-019-4148-4

**Published:** 2019-05-22

**Authors:** Antônio José Lana de Carvalho, Hyster Martins Ferreira, Eliza Fernanda Borges, Laerte Honorato Borges Junior, Ana Laura Teodoro de Paula, Wallisen Tadashi Hattori, e Vivian Mara Gonçalves de Oliveira Azevedo

**Affiliations:** 1 Graduate Program in Health Science, Faculty of Medicine, Federal University of Uberlândia, 1720 - Pará Ave., Umuarama, Uberlândia, 38405-320 Brazil; 2 Home Care Service, Hospital of Clinics of Uberlândia, Federal University of Uberlândia, 1720 - Pará Ave, Umuarama, Uberlândia, 38405-320 Brazil; 3 Institutional Program for Voluntary Scientific Initiation, Medical School, Federal University of Uberlândia, 1720 - Pará Ave., Bloco 2U, Umuarama, Uberlândia, 38402-022 Brazil; 4 Department of Public Health, Faculty of Medicine, Federal University of Uberlândia, 1720 - Pará Ave., Umuarama, Uberlândia, 38405-320 Brazil; 5 Faculty of Physical Education and Physical Therapy, Federal University of Uberlândia, 1286 - Benjamin Constant St., Nossa Sra. Aparecida, Uberlândia, 38400-678 Brazil

**Keywords:** Home care, Health service effectiveness, Chronic disease

## Abstract

**Background:**

Technological advances in health care currently provide better care conditions and have increased survival rates of premature infants, along with increasing the life expectancy of chronically ill children. In this context, the home care service has emerged as an effective tool for the treatment of this group of children. Thus, this preliminary study aimed at evaluating the effectiveness of the Home Care Service (HCS) with regard to pediatric care.

**Methods:**

A cross-sectional study was performed through a medical record analysis of a tertiary hospital in Minas Gerais/Brazil. Two groups were compared: 36 patients from the HCS (home group) and 13 patients hospitalized with an indication for home care (hospital group). To analyze the effectiveness of HCS, we evaluated the number of readmissions, infection rate, number of procedures, and optimization of beds.

**Results:**

The hospital group presented 6.04 times more infections and was submitted to 6.43 times more procedures. The home group presented lower readmission rates; with 41.66% of children studied not being readmitted and 76.19% of those who needed readmissions did so after more than 30 days from hospital discharge. HCS optimized hospital beds and allowed, over five (5) years, the hospitalization of around 102 patients in the hospital studied.

**Conclusion:**

In this preliminary study, HCS reduced the number of procedures and infections compared to hospitalized patients. Moreover, HCS presented lower readmission rates and optimized hospital beds, which could be considered an indication of effectiveness.

## Background

Technological advances in the treatment of health problems over recent years have provided better care conditions and increased survival of prematurely born children, as well as an increase in life expectancy and a decrease in child mortality [1], which provides an improvement to chronic health conditions throughout the world [2].

However, we conceptualized children with chronic health conditions of up to 1 year as a limiting and evolving condition, characterized by loss of locomotion capacity and loss of social relationships. In addition to the need for technological devices, the use of controlled and continuous medications, with gastrointestinal problems, mainly related to deglutition; psychological and cognitive alterations, dependent on neuropsychomotor stimulation, in addition to specialized educational and health services [3].

Several countries have tried to adapt to this new demand for offered care, due in particular to issues of economic viability [4]. This has strengthened the emergence of new strategies and mechanisms for health care, such as Home Care Services (HCS), which combines technological and scientific resources present in the hospital with the family environment [5]. The domicile, owing to its humanizing characteristic and the demographic and epidemiological profile of the population, is a place with potential to expand and qualify care processes [6].

The development of HCS around the world has been accompanied by demographic and epidemiological changes [7] and is related to reduction of risks of infections [8], humanization of care and quality of life, greater involvement of relatives with patient disease, tightening of health staff/patient/family relationship [9], reduction of costs, increased turnover of hospital beds with bed management, de-hospitalization [10], lower rates of clinical worsening and acute complications, lower demand for emergency and emergency services, lower rates of re-hospitalization [11], implementation of palliative care [12] and effective prevention, promotion and recovery of health actions [13].

Despite the expansion of HSC in several countries, there is still a need to investigate whether this type of service is as effective as the service provided in the hospital environment, this was performed using four primary variables: quantitative infections, number of readmissions, optimization of hospital beds and the number of procedures [8]. Therefore, this study had as its objective to evaluate the effectiveness of a Home Care Service, regarding pediatrics care [7, 14].

## Methods

### Population

This preliminary study included 57 pediatric patients with chronic health conditions, restricted socialization and limited contact with the external environment, low mobility, frequent exacerbations and locomotion restrictions, as well as belonging to type 2 (HC2) and type 3 (HC3) home care.

In Brazil, there are three types of home care: HC1, HC2 and HC3. The HC1 modality is composed of children who require minimal and less frequent multiprofessional interventions, who have health problems compensated by receiving care through their caregivers, and their health supervision is carried out by Basic Health Units (BHU) [15].

In the HC2 modality, patients with acute or chronic acute degenerative diseases are included, and who require constant and continuous care. They could be premature or of low weight, undergoing drug and non-parenteral infusion or in palliative care. In the HC3 modality, they present the same clinical conditions identified for the HC2 modality, but require continuous multiprofessional supervision, requiring mechanical ventilation, performing complex procedures, parenteral nutrition and blood transfusion, demanding constant home monitoring [16].

Patients were divided into two groups for comparison and the effectiveness analysis:

### Hospital group

The hospital group comprised of the convenience analysis containing 13 patients, that is all children with an indication for home care, since the beginning of the first indication of hospital discharge for home care maintenance in the institution where this study was carried out (from 2007 to 2016). For some reason they remained in the hospital for more than 30 days after the indication for home care service because of a social context or to finalize the training of home caregivers. This indication for hospital discharge was identified from health professional records, which indicated hospital discharge and continuation of home care, and the date of the discharge report was the beginning of the data collection process until the clinical outcome, transfer and consequential admission to the HCS.

### Home group

**Fig. 1:**
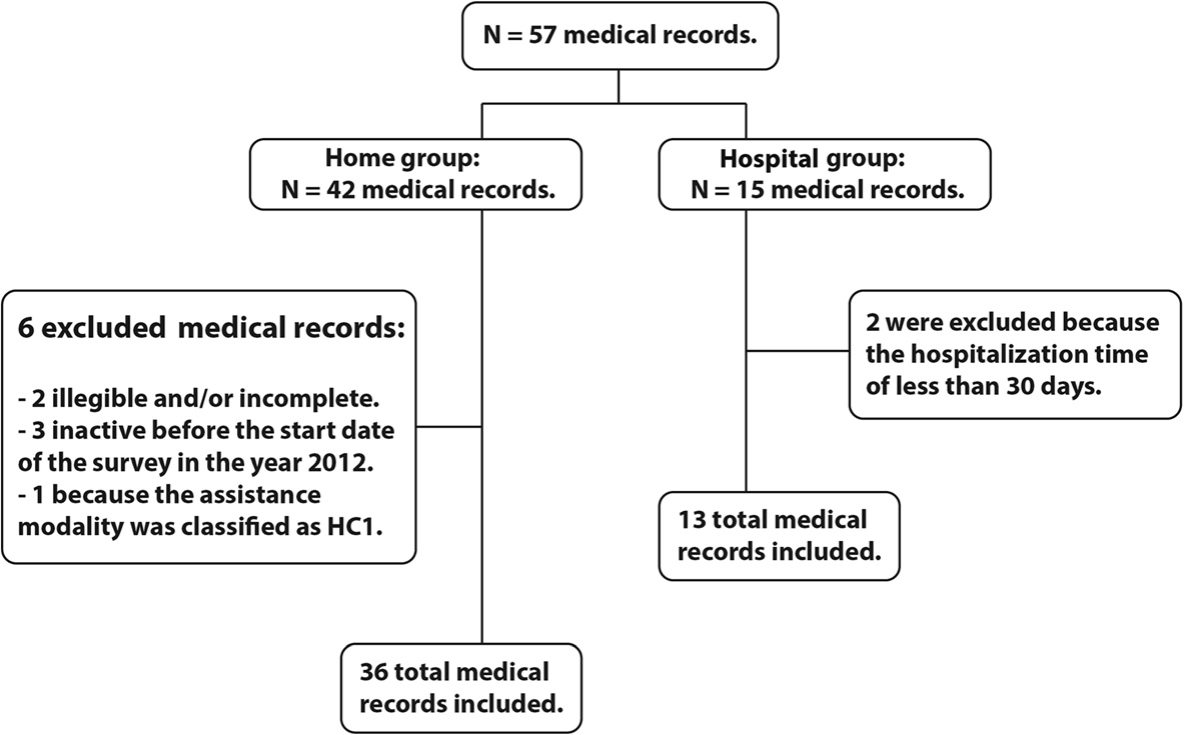
Study Flow chart

A total of 36 HCS-assisted children were selected over the 5-year interval (January 01, 2012 to December 31, 2016). Patients with a home care interval of less than 30 days were excluded in order to match the minimum adopted care interval as a criterion in the hospital sample (Fig. 1).

Figure 1 Number of patients included and excluded in the study and their respective groups.

Concerning the 49 children included in this study, 36 were assisted through HCS and 13 through the hospital service. The characteristics of the evaluated population are presented on Table 1.

**Table 1:** Sample characteristics: Patients in home care assisted by a home care service (HCS) and patients hospitalized in a hospital service. Brazil, 2012–2016

Variables	Modality of assistance (n / %)		*p*-value
	Home group *n* = 36)	Hospital group (*n* = 13)	
Sex^a^			0.989
Male	25 / 30.56	9 / 69.23	
Female	11 / 69.44	4 / 30.77	
Dependence degree^b^			0.561
Wanders	2 / 5.56	1 / 7.60	
Wheelchair user	1 / 2.78	1 / 7.69	
Bedridden	33 / 91.67	11 / 84.62	
Classification by type of care^b^			0.663
HC2	7 / 19.44	1 / 7.69	
HC3	29 / 8.56	12 / 92.31	
Medical devices^c^			–
Oxygen therapy	9 / 25.00	8 / 61.5	
IMV^d^	19 / 52.78	9 / 69.2	
IMV Intermittent	8 / 22.22	3 / 23.0	
NIMV^d^	0 / 0.00	0 / 0.00	
NIMV Intermittent	2 / 5.56	0 / 0.00	
GTM	31 / 86.11	12 / 92.3	
PVC	0 / 0.00	1 / 7.69	
Tracheostomy	27 / 75.00	10 / 76.9	
Colostomy	1 / 2.78	1 / 7.69	
Tenckoff catheter	1 / 2.78	1 / 7.69	
Main Diagnosis^c^			–
Pulmonary disease	4 / 11.11	0 / 0.00	
Neurological disease	16 / 44.44	7 / 53.85	
Genetic syndrome	12 / 33.33	2 / 15.38	
Others	4 / 11.11	4 / 30.77	
Age Group^b^			0.465
Infants (29 days to 12 months)	13 / 38.2	2 / 15.38	
Toddlers (1 to 3 years old)	14 / 41.2	6 / 46.15	
Kindergarten children (4 to 5 years old)	3 / 8.8	1 / 7.69	
School children (6 to 12 years old)	6 / 17.6	4 / 30.77	

*HC2* Home care type 2, *HC3* Home care type 3, *IMV* Invasive mechanical ventilation, *NIMV* Noninvasive mechanical ventilation, *GTM* Gastrostomy, *PVC* Permanent venous catheter

^a^Chi-square Independence test applied to the variable and Modality of assistance

^b^Fisher Exact test applied to the variable and Modality of assistance

^c^No statistical association was possible due low frequency in some categories

^d^continuous use

Table 1 Sample characteristics: patients in home care and patients hospitalized. Brazil, 2012–2016.

### Procedure

The study was designed as a cross-sectional study. The collection and selection of medical records were performed in both the medical records and inactive files, analyzing all pediatric patients aged between 29 days and 11 years, 11 months and 29 days of age. This was seen as an adequate age range for admission to the pediatric ward of a tertiary level university hospital and into the HCS program of the same institution [15].

Data collection was performed using a spreadsheet structured from a table of indicators that contained four main variables for the evaluation of HCS effectiveness: readmissions, number of infections, optimization of hospital beds and procedures performed (laboratory and imaging examinations).

We analyzed epidemiological profiles, degree of dependence, classification of the type of care (HC2 and HC3), main diagnosis, care category (neuromuscular disease, genetic syndrome, pulmonary disease, cerebral palsy, spinal cord injuries and others), need for mechanical ventilation or device used.

For the analysis of effectiveness, the study contemplated the pairing of the two groups, and the observations made in the first group were paired with those of the second, guaranteeing an equivalent composition.

Concerning the number of procedures, we analyzed all laboratory and imaging exams performed during the study period. Among the laboratory tests, the most notable are urine exams (Abnormalities of urinary sediment and urine culture), blood tests (blood culture, biochemistry, venous and arterial blood gas analysis, Troponin Activity Time (TAP) and central venous catheter aspiration), tracheal secretion, central venous catheter tip, ocular secretion swabs and wounds, faeces (parasitological and coproculture), aspirate liquid acetic (cytological and culture of peritoneal fluid). In addition to the aforementioned examinations, imaging exams were performed, those being x-ray, electrocardiograms, echocardiograms and ultrasonography.

### Data analysis

Descriptive analyzes were carried out with the use of absolute frequency and percentage for categorical and ordinal variables, maximum and minimum values and mean for metric variables. Infection rates, bed optimization, re-hospitalization and procedures were calculated considering the total number of records divided by the total number of days of hospitalization (home or hospital) of each group. Thus, the rate found did not depend on the number of participants in each sample, but on the occurrence of these events within their respective groups.

This study was approved by the Ethics Committee on Human Research of the Federal University of Uberlândia (approval number: 1823938).

## Results

For the analysis of readmissions, we verified how many days after the date of admission into the HCS these patients were submitted to the first hospital readmission, in a time interval before and after 30 days [16]. We also took as a reference the total number of days of re-hospitalization (Table 2).

**Table 2:** Occurrences of hospital readmissions (exclusive to the home group), assisted by a Home Care Service. Brazil, 2012–2016

Hospital readmissions	Home group (*n* = 36) n / %
Patients who never were re-hospitalized	15 / 41.66
Patients re-hospitalized	21 / 58.33
First readmission, between 10 and 30 days after admission to HCS	5 / 23.81
Readmission after 30 days of admission in HCS	16 / 76.19
Readmission after 3 months after admission in HCS	13 / 24.07
Total days in readmission/total days in HCS	930 / 2.91

55 cases of readmissions for 36 patients included. Average of 11.92 days of readmission for a total of 930 days

Infection, outcome and procedure rates were calculated considering the total number of records divided by the total number of days of hospitalization (home or hospital) of each group. Thus, the rate found did not depend on the number of participants in each sample, but on the occurrence of these events within their respective groups (Table 3).

**Table 3:** Number of procedures (laboratory tests and imaging tests), number of infections, days of hospitalization and clinical outcome of patients in home care. Brazil, 2012–2016

Variables	Modality of assistance			
	Home group (*n* = 36)	medium (máx/mín)	Hospital group (*n* = 13)	medium (máx/mín)
Number of Procedures				
Total	432	9 (56 / 1)	228	12 (61 / 3)
Laboratory	367	7 (52 / 1)	211	12 (60 / 2)
Imaging	65	1 (10 / 1)	17	1 (5 / 1)
No. of procedures / day	0.014	–	0.09	–
No. of procedures / 30 days	0.40	–	2.8	–
No. of procedures / 60 days	0.80	–	5.6	–
Infections				
Yes (n/%)	17 /28.33	–	37 / 90.24	–
Total no. of infections per patient	0.47	–	2.84	–
No. of infections per day	0.0005	–	0.01	–
No. of infections for 30 days	0.016	–	0.4	–
No. of infections for 60 days	0.03	–	0.8	–
Total days in hospital	31.922	656	2630	104.50
Outcome				
Remains in hospital (n/%)	21 / 58.33	–	13 / 100	–
Clinical discharge (n/%)	5 / 13.89	–	0 / 0.00	–
Hospital death (n/%)	9 / 25.00	–	0 / 0.00	–
Home death (n/%)	2 / 5.56	–	0 / 0.00	–
Administrative discharge (n/%)	1 / 2.78	–	0 / 0.00	–

Table 3 Comparative analysis of procedures, infections, days of hospitalization and clinical outcome. Brazil, 2012–2016.

Regarding the optimization of beds, the following analysis and subsequent calculation were used in order to verify the sum of the total days of hospitalization of home group patients, between 01/01/2012 and 12/31/2016. There were found to be 31,922 days of hospitalization in this service; this number divided by the average number of hospitalization days per patient in the pediatric ward, arrives at 9.80 days (Source: Hospital information management and statistical sector). Thus, we reached 3257.35 days available for HCS. By dividing this number (3257.35 days) by the number of active beds (32 beds) in the pediatric unit, it would be possible, over five (5) years, to hospitalize around 102 patients in the hospital studied.

## Discussion

In order to evaluate the effectiveness of a Home Care Service, comparative and equivalent to that offered by in-hospital service, from four variables, those being the number of infections, the number of readmissions, the optimization of hospital beds and the number of hospitalizations procedures. The study showed that HCS has low infection rates, procedures and readmissions, while optimizing hospital beds, which all can be considered as an indication of its effectiveness.

Regarding the number of infections, the hospital group presented an infection rate 6.04 times higher than the home group. A study carried out in a French university hospital showed that infections were the main cause of death in hospitalized patients [17]. The length of hospital stay is a contributing factor for occurrence of infections related to hospital or nosocomial resistance [18]. A review conducted in 2014 found that antimicrobial resistance could cause the death of up to 10 million people per year by 2050 [19]. Indeed, measures for prevention of Health Care Related Infections (HCRI) should be adopted in all health care settings, either in the hospital setting, in chronic care facilities or in home care, in light of this growing threat to global public health [20].

In order to verify optimization of hospital beds, according to the calculation demonstrated previously. HCS stands out for its contribution to the optimization of hospital beds. It is an effective care tool that contributes to reducing the need for the hospitalization of patients with high utilization rates of hospital services [21].

Regarding the procedures, the results showed that patients in the hospital group were submitted to 6.43 times more procedures than patients from the home group in the same time interval, this result is very similar to the infection rate. In fact, in recent decades, public expenditures on health has increased exponentially, mainly due to the excess number of laboratory and radiographic examinations requested by doctors, performed in an inadequate and/or unnecessary way [22]. Since 1998, 95% of the examinations performed are repetitively requested, and as such contribute to increasing the cost of care for patients admitted to hospitals [23]. Although the tests are useful for diagnosis, it is necessary to consider the high financial cost involved for health services [24]. According to an English systematic review, with 109 articles aimed at reducing the use of laboratory tests by physicians, showed that educational measures were more effective when it came to reducing their use [25]. In Brazil, the Ministry of Health did not set a limit for exams [26]. In light of the aforementioned, one notes that HCS is a promising instrument in terms of the reduction of health costs, when referring to the relevance of procedures.

In terms of the 36 home care patients who participated in this study, 15 (41.66%) were never re-hospitalized and 21 (58.33%) had one or more readmissions, and 5 patients returned to the hospital within 10 to 30 days (23.81%), 16 patients after 30 days (76.19%) and 13 after 3 months (24.07%) from the date of hospital discharge. We point out that all re-hospitalizations occurred in the same reference hospital for the care of these patients, according to an agreement made between the Public Prosecutor’s Office, family members and the health institution, during the process of de-hospitalization. In 2002, a Spanish study identified low readmission rates of 10, 28 days, and 3 months of 3.6, 9.7 and 13.5%, respectively [27]. In fact, hospital readmissions are a frequent problem in health institutions. They are also an indicator of quality of care, based on the analysis of people admitted for the first time to the hospital within 30 days after discharge [16]. This indicator assesses the progressive capacity of the service provider in terms of helping patient recovery to happen as effectively as possible, with regard to patient care after hospital discharge [28]. In fact, there is a dearth of data in medical literature for the evaluation of early hospital readmissions in pediatrics with a specific focus on home care.

The study, however, contains limitations for its retrospective treatment and data collection, both performed through the analysis of medical records, which at times present incomplete and/or illegible records. As HCS has recent legal norms for implementation and regulation, there are few studies regarding the effectiveness of HCS in Brazil, addressing pediatric patients. Another complicating factor is the lack of parameters for analysis, where the quantity of exams is considered to be in excess, since the Ministry of Health does not establish any normative indication.

## Conclusions

This preliminary study showed that HCS-assisted patients underwent fewer procedures and demonstrated a significant reduction in their use, in addition to a low number of infections compared to hospitalized patients. Moreover, HCS presented lower readmissions rates and optimized hospital beds, which could be considered an indication of effectiveness.

## Abbreviations

BHU: Basic Health Units
GTM: Gastrostomy
HC1: Home Care type 1
HC2: Home Care type 2
HC3: Home Care type 3
HCRI: Health Care Related Infections
HCS: Home Care Service
IMV: Invasive Mechanical Ventilation
NIMV: Noninvasive mechanical ventilation
PVC: Permanent venous catheter
TAP: Troponin Activity Time
